# Anatomical circuits for flexible spatial mapping by single neurons in posterior parietal cortex

**DOI:** 10.1038/s42003-025-08596-6

**Published:** 2025-09-09

**Authors:** Bashir Ahmed, Hee-kyoung Ko, Maria Rüsseler, Jackson E. T. Smith, Kristine Krug

**Affiliations:** 1 Department of Physiology Anatomy and Genetics, University of Oxford, Oxford, UK; 2 Institute of Biology, Otto-von-Guericke-University Magdeburg, Magdeburg, Germany; 3 Leibniz-Institute for Neurobiology (LIN), Magdeburg, Germany; 4 Centre for Behavioral Brain Sciences (CBBS), Otto-von-Guericke-University Magdeburg, Magdeburg, Germany; 5 Present Address: Department of Psychology and Clinical Language Sciences, Reading University, Reading, UK

**Keywords:** Decision, Motion detection, Neural circuits

## Abstract

Primate lateral intraparietal area (LIP) has been directly linked to perceptual categorization and decision-making. However, the intrinsic LIP circuitry that gives rise to the flexible generation of motor responses to sensory instruction remains unclear. Using retrograde tracers, we delineate two distinct operational compartments based on different intrinsic connectivity patterns of dorsal and ventral LIP. These connections form an anatomical loop with a sensory-like, point-to-point projection from ventral to dorsal LIP and an asymmetric, widespread projection in reverse. In neurophysiological recordings, LIP neurons exhibit motor response fields spatially distinct from their sensory receptive field. Different associations of motor response and receptive fields in single neurons tile visual space. Ventral LIP neurons tend to have motor response fields distant from their sensory receptive fields. This circuit provides the neural substrate to generate the dynamic processes for flexible allocation of attention and motor responses in response to salient or instructive visual input across the visual field.

## Abstract (web summary)

Neuronal tracing reveals unbalanced circuits between two distinct operational compartments in primate lateral intraparietal area (LIP), which underpin single LIP neurons with spatially different sensory and motor receptive fields mapped neurophysiologically.

## Introduction

Posterior Parietal Cortex (PPC) constitutes a hub of brain areas which compute sensorimotor transformations and contribute to cognitive functions like action selection, spatial awareness, sensory evaluation and directing attention in primates^1–3^. One of the most studied PPC areas is lateral intraparietal area (LIP), which has been afforded central roles in attentional processing, decision-making, visual categorisation and oculo-motor planning^4–8^. Cyto-architectonic and myelin-architectural studies have shown that LIP can be subdivided into a dorsal (LIPd) and ventral part (LIPv)^9,10^. The connectivity with other cortical areas differs between LIPd and LIPv^9,11–14^. LIPv provides strong feed-forward connections to oculomotor areas such as the frontal eye fields (FEF) and the superior colliculus (SC) as well as receiving input (amongst others) from dorsal visual area V5/MT, a connection missing for LIPd. In contrast, LIPd receives input from ventral stream visual areas and has unique connections with the cingulate cortex and the dysgranular insular cortex. The differential profile of laminar terminations for the projections from area FEF shows that LIPd receives feedforward input from FEF while LIPv receives feedback projections^15^. These findings taken together with inactivation studies suggest that LIPd and LIPv should also differ in their functional roles^16^. Despite these differences, the intrinsic circuitry within LIP remains unclear.

Area LIP lies on the posterior-lateral bank of the intraparietal sulcus. From the earliest neurophysiological studies, neurons in area LIP have been characterised by their “motor field” (MF), often also termed “response field”, which describes the preferred direction and amplitude of an upcoming saccade into the MF^17^, even in the absence of a visual target stimulus^18,19^. In other studies, LIP neurons were shown to respond to visual stimuli in their sensory “receptive field” (RF)^20^ and can be tuned to features like direction of motion or binocular disparity^21,22^; RFs are re-mapped to their expected location *before* an upcoming saccade and RFs are also modulated by their behavioural significance^3,23^. Visual RFs have usually been described as co-localised with MFs and to represent information about saccade targets^19,24^. Our results presented here show that this is one option among a range of actual spatial configurations.

Due to its strong activation by visual stimuli and planned saccades, it is not surprising that LIP is crudely topographically organised, at least in LIPv, with a preference for the contralateral visual field^9,25,26^. However, in the literature, the type of neuronal responses reported and their organisation in LIP differ, usually depending on whether an animal carried out a (saccade) task or fixated a target or cells were recorded in anaesthetised animals. Also, initial screening for LIP neurons (whether during saccades or during passive viewing of visual stimuli) varied between studies. More recently, two intersecting topographic maps (of visual attention and saccade planning) have been suggested for LIPv^27^. The alignment between the two maps and especially how they intersect in single neurons, which is critical to how LIP neurons compute sensorimotor transformations, is one of the questions we address in this study.

## Results

**Fig. 1:**
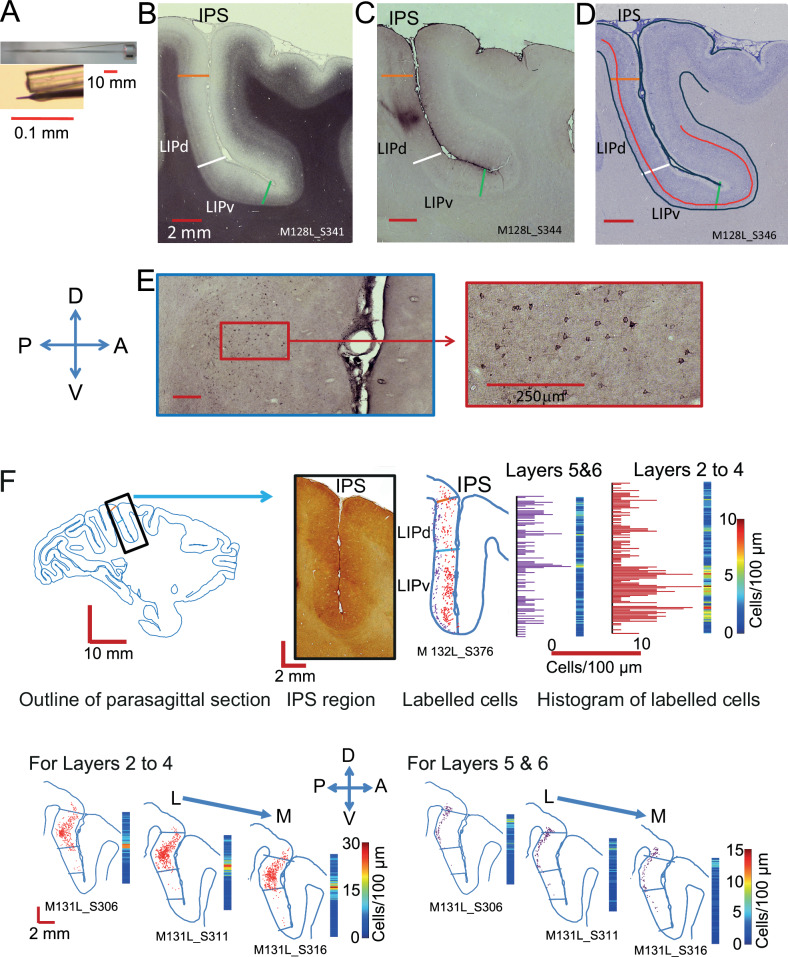
Tracer injections and analysis. **A** Injections were placed via a glass capillary tube filled with tracer which was glued to a tungsten electrode for recording neural activity (reproduced with permission^28^). **B**,** D** Parasagittal sections through the intraparietal sulcus. **B** This section was stained for myelin using the Gallyas method. LIPv is identified histologically by the more dense, extended intracortical myelin, LIPd by the two bands. **C** Nearest parasagittal section (to B) showing the CTb injection site in LIPd, taking a posterior approach. For LIPv, we took either a posterior approach (two animals) or a dorsal approach (one animal). **D** Nearest Nissl-stained section (to **C**) identifying the border (red line) between layers IV and V to be able to distinguish potential differences in the pattern of connectivity in supra- and infragranular layers. **E** Section stained for CTb shows the retrogradely labelled neurons. The high-resolution image (right) reveals the shape and granulated fill of individual neurons, which were the criteria for accepting a neuron as labelled. **F** Method for the data transformation of CTb labelled cells from an individual parasagittal sections into 3D maps of intrinsic LIP connectivity using two example injections. Top: Labelled cells were converted to density measures in 100 µm bins along the dorso-ventral axis, separately for supragranular (layers 2–4) and infragranular layers (5 and 6) (example M132L after LIPv injection). No smoothing across bins took place. Bottom: Then, data were aligned section-by-section along the medio-lateral extent of LIP (example M131L after LIPd injection). A – anterior, P – posterior, L – lateral, M – medial, D – dorsal, V – ventral.

To investigate the intrinsic connectivity of cortical area LIP, we placed single small, focal injections of retrograde tracers Cholera Toxin subunit b(CTb) or Fluorogold (FG) into LIP in one hemisphere of six Rhesus macaques (*Macaca mulatta*) (Fig. 1). After 2–12 days survival, animals were transcardially perfused and tissue histologically processed. A one-in-five series of parasagittal sections (50 µm) of the injected hemisphere was stained for myelin to ascertain LIPv and LIPd borders (Fig. 1B). All LIPv/LIPd borders in the figures are derived from interleaved sections stained for myelin, with LIPv showing the distinct pattern of dense myelination in contrast to two lighter, discernible bands in LIPd. Retrogradely labelled cell bodies within LIP were analysed using Neurolucida in another one-in-five series of the same hemisphere (Fig. 1C, E). Labelled cells were only counted when the shape of the cell body was clearly identifiable and its label was black and granulated, as is typical for CTb staining.

### LIPv neurons send point-to-point projection to a single site in LIPd

**Fig. 2:**
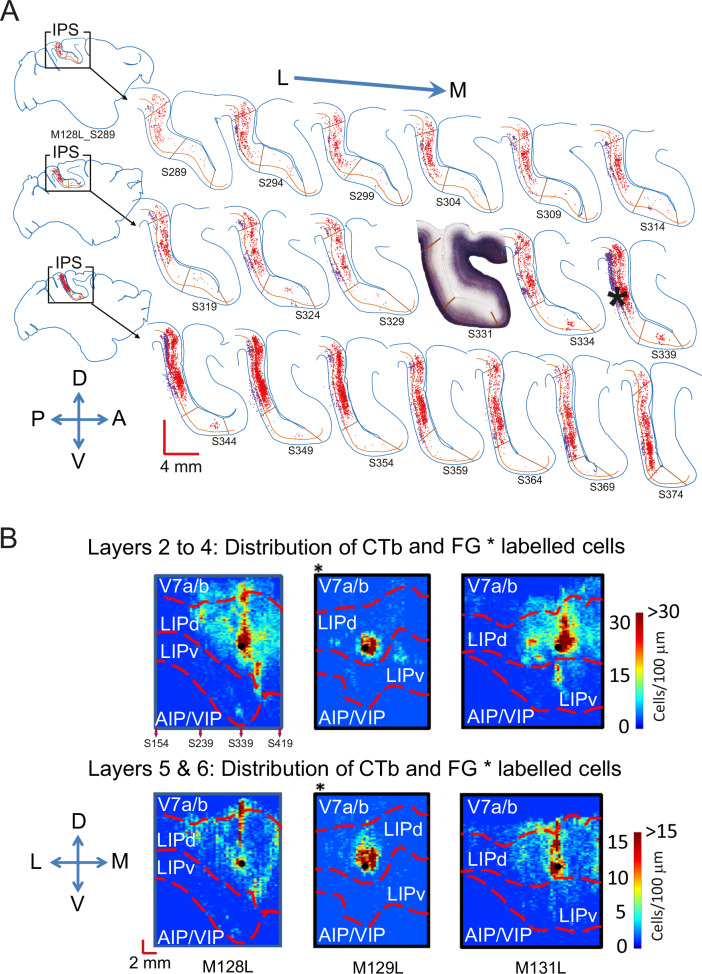
Neurons in LIPd receive topographic input from a single site in LIPv but widespread input from within LIPd. **A** A one-in-five series of parasagittal sections shows the distribution of labelled cells after a CTb injection into LIPd in the left hemisphere of one animal (M128L). Retrogradely labelled neurons can be found throughout the layers and the extent of LIPd, but there is only one clear cluster of labelled cells in LIPv that project to the injection site. The densest label in LIPd is medial to the injection site. The inset section shows the myelin definitions of LIPv and LIPd from an alternate series. Red cells are in supragranular layers I-IV, purple cells in infragranular layers V-VI; * denotes the injection site. See Fig. 3A as well as Supplementary Figs. 2 and 3 for histological images before annotation and further Gallyas sections. **B** The plots summarise the 3D-pattern of label (dorso-ventral; medio-lateral; supragranular and infragranular layers) across all three animals with a tracer injection into LIPd (denoted as filled black dot). M129 (*) received an injection of the retrograde tracer Fluorogold, M128 and M131 of CTb. All three animals show an LIPd-intrinsic, wide-spread network of neurons that project to the injection site in area LIPd itself. They also all show a single cluster of labelled cells in LIPv indicative of a topographic input from LIPv to LIPd. For M128L, we show section numbers in the density map relating to the sections in A. L – lateral, M – medial, D – dorsal, V – ventral, A – anterior, P – posterior.

**Fig. 3:**
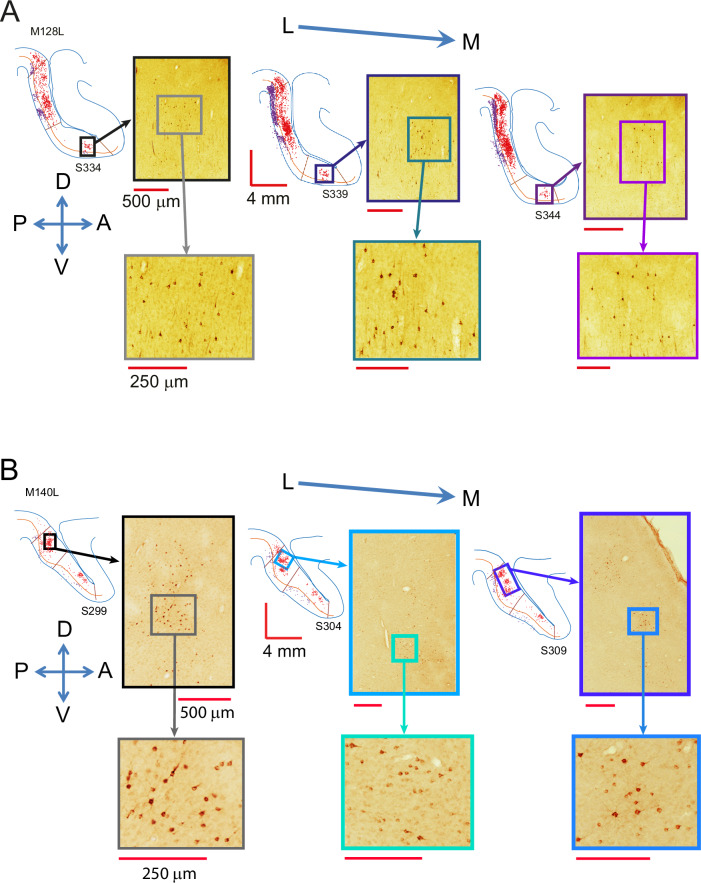
Histological images of clusters of labelled cells. **A** Histological sections showing the retrogradely labelled cells of the cluster in LIPv for the brain presented in Fig. 2A after an injection in LIPd, at low and high magnification. **B** Histological sections showing the retrogradely labelled cells of a cluster in LIPd for the brain presented in Fig. 4A after an injection of retrograde tracer in LIPv.

First, we analysed three brains (M128, M129, M131) with an injection placed in LIPd (Fig. 2). A small injection in area LIPd retrogradely labelled neuronal cell bodies throughout the medio-lateral and dorso-ventral extent of LIPd itself (Fig. 2A). These labelled cells have synaptic connections to the injections site. The extent of labelled neurons in LIPd is comparable to the long range of intrinsic connectivity within extrastriate visual areas V5/MT or TE^28,29^. In contrast, we find only a single cluster of labelled neurons in LIPv which projects to the injection site in LIPd (Figs. 2, 3A). We analysed the three-dimensional pattern of labelling by projecting the labelled cell data from each section onto a line. We did this separately for upper and lower layers (Fig. 1F), because supragranular and infragranular layers have usually different connectivity^30,31^. The labelled cell densities from each section were then aligned across all the analysed parasagittal sections through LIP using landmarks and area borders (Fig. 1F, Supplementary Fig. 1) to compare the pattern of label across the different animals. Clustering of labelled cells tended to be stronger in the dorso-ventral plane of the injection site and medial to the injection site (Fig. 2B). But the density of labelled cells drastically decreased near the LIPd/LIPv border. Although retrograde tracer Fluorogold (FG) labelled neurons less densely, the observation of a single cluster in LIPv projecting to LIPd is confirmed by the data from all three brains regardless of the retrograde tracer (CTb or FG) used (Fig. 2B; see also a reconstruction in Supplementary Video S1). This pattern is observed in supra- and infragranular layers, although more weakly in the latter. Like the bulk of the LIPd label, this cluster appears near the same dorso-ventral line (M128, M131) or immediately medial to the injection site (M129) and is more pronounced in the supragranular layers. This pattern of connectivity reveals a topographic, point-to-point input from LIPv to LIPd. This is the type of sparse, ordered projection one would see in early to mid-level sensory areas in primates, as for example from V1 to V2 or to V5/MT, and which underpins the neurophysiological responses defining the spatially precise sensory receptive fields in these areas^32–35^.

### LIPv neurons receive projections from multiple distinct cell clusters in LIPd

**Fig. 4:**
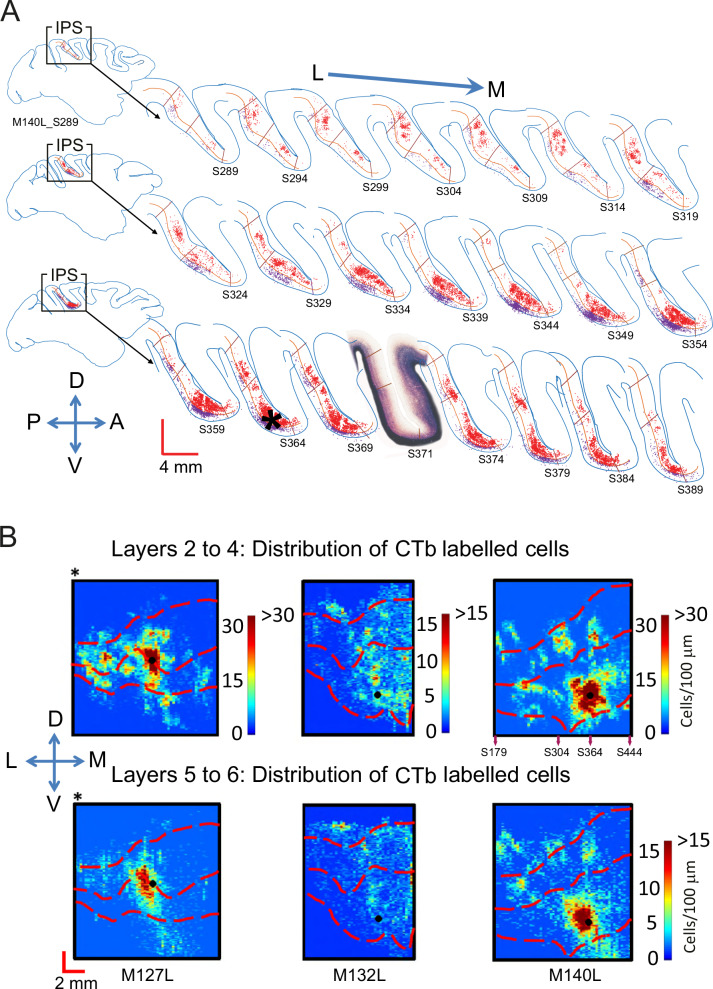
Neurons in LIPv receive wide-spread input from within LIPv and from several cell clusters in LIPd. **A** A one-in-five series of parasagittal sections shows the distribution of labelled cells following a CTb injection in LIPv (M140L). Retrogradely labelled neurons can be found in supra- and infragranular layers across LIPv. In LIPd, several clusters of labelled cells are seen throughout the medio-lateral extent of LIPd. The inset section shows the myelin definitions of LIPv and LIPd from an alternate series. Red cells are in supragranular layers 2–4, purple cells in infragranular layers 5 + 6 with largely similar results; * denotes the injection site. **B** Plots summarise the 3D-pattern of label across all three animals with a tracer injection into LIPv (at the LIPd/v border for M127L *). All three animals show an LIPv-intrinsic wide-spread network of neurons that project to the injection site as well as multiple clusters of labelled cells in LIPd projecting to LIPv. For M140L, we show section numbers underneath the density map related to the sections depicted in A. See Supplementary Fig. 4 for a histological image of M132L before annotation. L – lateral, M – medial, D – dorsal, V – ventral, A – anterior, P – posterior.

In three further animals, we placed CTb injections into LIPv (M127, M132, M140). The pattern of label in the example hemisphere in Fig. 4A shows that a single point in LIPv receives inputs from neurons all across LIPv, with the highest density of labelled neurons found around the injection site. There was again evidence of strong connectivity along the dorso-ventral axis in line with the injection site. In stark contrast to the sparse, topographic input from LIPv to LIPd shown in Fig. 2, LIPv received more widespread inputs from several clusters of labelled cells across the medio-lateral extent of LIPd, mapping many points across LIPd to one point in LIPv. This was the case whether the injection was confined to LIPv (M132, M140) or also was partly including LIPd at the border (M127) (Figs. 3B, 4B). Overall, the pattern of connectivity was comparable in supra- and infragranular layers, but some clusters of labelled cells were missing or weaker in the infragranular layers (Fig. 4A, B).

Thus, the LIPv-LIPd connectivity forms a circuit that potentially can map one location in topographically organised LIPv to many other locations represented in LIP in a recurrent network. The functional nature of the LIP map(s) is under debate^27^. Next, we investigated this question at the neurophysiological level of single LIP neurons.

### LIP neurons have spatially separate motor response and sensory receptive fields

**Fig. 5:**
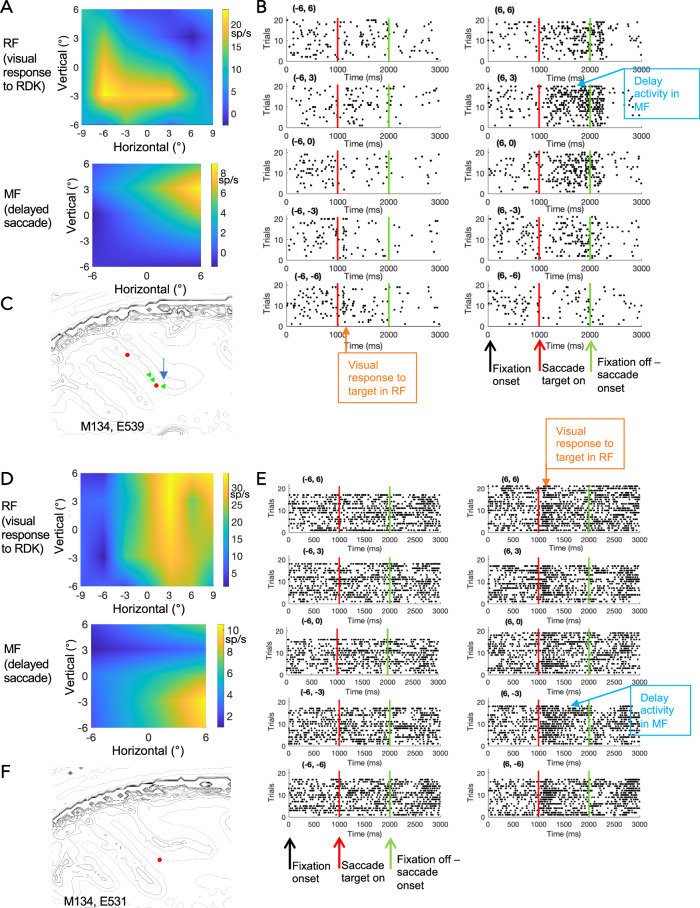
The RF and MF of example LIP neurons. **A**–**C** Data from one LIP neuron. **A** This example cell has a visual receptive field (RF) as mapped with a RDK stimulus (top) that is spatially distinct from its  motor response field (MF) (bottom) in a delayed saccade task. Scale is relative to central fixation at 0°,0°. See Supplementary Fig. 5 for unsmoothed representations of the RF and MF maps. **B** The raster plots for the delayed saccade task for the MF shown in (**A**). Each plot represents a saccade target position. Degrees of visual angle from central fixation are given above the plot. Each line represents a trial with dots as individual spikes. The neuron shows a brief response to the onset of the saccade targets (a dot) at positions -6°,-3° and -6°,-6°, in the region where the sensory RF was localised to in the separate mapping experiments with an RDK (see **A**). But the delay activity was recorded while the animal was waiting to carry out a saccade to targets at +6, +3 and +6, 0. **C** Grey outlines highlight tissue boundaries derived from the structural MRI obtained from the individual animal. The coloured dot gives the projected location of the recorded example cell depicted in (**A**, **B**); the colour identifies the type of MF-RF relationship (green: MF and RF located in different visual hemifields). Where we recorded multiple cells in the same parasagittal plane, an arrow points to the example neuron. **D**–**F** Data from another LIP neuron. Same conventions as in (**A**–**C**). **F** The coloured dots give the projected location of the recorded cells, the colour identifies the type of MF-RF relationship (red – both contralateral, green – across different hemifields). More examples can be found in Supplementary Fig. 6.

LIP combines both sensory and motor inputs from different sources with distinct functional topographic maps. We investigated how these maps might intersect in single neurons. We tested the neurophysiological response maps for 111 single LIP neurons in two macaque monkeys (41 from M133, 70 from M134), for which we could determine a motor response field (MF) and a visual receptive field (RF) online during the experiment. We systematically mapped their MF with a delayed saccade task and their visual RF with a moving random dot kinetogram (RDK) (Fig. 5). After carefully isolating single units offline and statistical analysis of stimulus and task related responses, 66/111 LIP neurons had a clear sensory receptive field (RF) (ANOVA/t-test *p* < 0.05), 60/111 LIP neurons showed a robust preference for a target location during the delayed saccade (MF) (ANOVA *p* < 0.05) and 45/111 LIP neurons had a significant RF and MF. Many neurons also showed clear tuning for direction of motion or binocular depth in their RFs as previously described^21,22^.

**Fig. 6:**
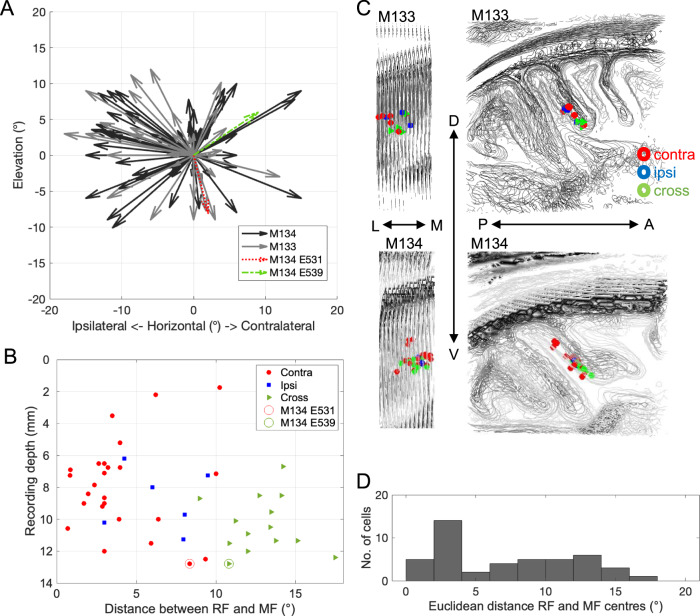
Distribution of MFs and RFs of LIP neurons. **A** For this figure, we projected all RF centres of LIP neurons to the centre of the graph (0°, 0°) and plotted the direction and relative distance of the MF centres as an arrow. This figure includes all LIP cells that we could map qualitatively online (*n* = 111). Some RF and MF centres are very close to each other, others quite far removed. As most RFs are found in the contralateral visual hemifield and about half of the MFs on the ipsilateral side, many arrows point to the left. Overall, a large range of different associations can be found, which tile the mapped visual field. Colour-coded are the examples from Fig. 5. **B** Recording depth (out of guide tube, 2 mm into the brain) was plotted against distance between RF and MF centres for LIP neurons with a significant RF and MF (*n* = 45). The colour and shape identify the type of MF-RF relationship (red circle: MF and RF in the contralateral visual hemifield; blue square: MF and RF ipsilateral; green triangle: MF and RF in different visual hemifields). **C** Spatial location of all LIP neurons with significant RF and MF for the two monkeys (M133, M134). LIP neurons with RF and MF across the two hemifields tend to be located more ventrally. Grey outlines depict tissue boundaries (extracted with Matlab using *contourslice*) for stacked parasagittal sections in a structural MRI volume centred on LIP. Views are either from posterior (left) or from the side (right). **D** Distribution of absolute distances between RF and MF centres for LIP neurons with a significant RF and MF (*n* = 45). In our sample, many LIP neurons have overlapping RFs and MFs but a considerable fraction can be as far apart as 12–18 degrees.

The distribution of RF and MF spatial response properties,  considered separately, matched those previously reported, with a preference for contralateral RFs but ipsilateral and contralateral MFs were more evenly distributed^9,18,26,36^. In many cases of cells with both RF and MF, we found the two were not spatially congruent (Fig. 5) and could be even in opposite hemispheres (see example in Fig. 5A–C). Thus, the firing rate of single LIP neurons showed responses to a visual stimulus in one part of the visual field as well as responses to the planning of a saccade towards another, distant location. When we compared the positions and distances of RF and MF centres for the recorded LIP neurons, we found that the possible associations between MF and RF tile the visual space we mapped experimentally (Fig. 6A). While many LIP neurons have RF and MF centres in the same visual map location, others can be quite far apart, up to 18 degrees (Fig. 6D).

Dual topographic maps (for saccade targets and sensory stimuli) with reversed map polarity have been proposed for LIPv^27^. If these mapped onto single neurons, the consequence would potentially be separated MFs and RFs in LIPv, as we describe above. The LIP neurons in our study were recorded with stereotactically placed vertical penetrations into area LIP. We carefully recorded the depth and the grey matter-white matter boundaries for each penetration, which allowed us to reconstruct the recording site of LIP cells in structural MRIs obtained from the individual animals (see Methods for details) (Fig. 6C). These reconstructions indicate a bias for neurons with RF and MF in opposite hemispheres to be found more ventrally in our recordings. While it is difficult to precisely place the LIPd/LIPv border in relation to our neurophysiological recordings without lesions and histology, we did compare the average depth of recording for LIP neurons with contralateral RF and MF (mean 8.2 mm ± 3.0, *n* = 25), ipsilateral RF and MF (8.8 mm ± 1.9, *n* = 6) and cross-hemifield RF and MF (10.3 mm ± 1.8, *n *= 14) for all neurons with offline quantitatively significant RF and MFs (*n* = 45). The statistical comparison showed a significant difference in recording depth between neurons with contralateral and cross-hemisphere RFs and MFs *(*two-sample t-test, *p* = 0.015) but not with the other group. We also found LIP neurons with a larger distance between RF and MF centres at greater recording depths (Spearman’s *r* = 0.29, *p* < 0.05, one-sided, *n* = 45) (Fig. 6B).

## Discussion

We show a distinct, asymmetric pattern of connectivity forming a loop between anatomically defined ventral LIP (LIPv) and dorsal LIP (LIPd). LIPv provides a sparse, point-to-point projection to LIPd, like one would see in early or mid-level sensory areas. In turn, multiple clusters of LIPd cells project to one point in LIPv, linking potentially many points to one map location. Neurons within either sub-compartment of LIP are widely interconnected. This circuitry forms the basis of processing and associating with each other the distinct sensory, motor and cognitive inputs coming into LIPv and LIPd from visual and prefrontal areas. Neurophysiological recordings demonstrated that the association of distinct sensory and motor planning signals is computed in single LIP neurons, which can have spatially separate visual RFs and saccadic MFs, especially in more ventral parts of LIP, which have previously been shown to be topographically ordered^25^. In sum, the neuroanatomical findings underpin a functional sensorimotor architecture that can link topographically organised, instructive visual stimuli to oculo-motor, attention or decision targets in single neurons.

The most striking feature of the intrinsic connectivity is the sparse, topographic input from LIPv to LIPd in stark contrast to the reciprocal, wide-spread connectivity from LIPd to LIPv and the widespread, within-compartment connectivity. The point-to-point pattern of connectivity for LIPv to LIPd is consistent in our data for all three animals and across two different types of retrograde tracer. For the more wide-spread return connections from LIPd to LIPv, we could place in two cases the injection exclusively into LIPv. However, the third animal with the injection at the LIPv/LIPd border confirms that as soon as LIPv is included in the injection site, we see wide-spread input from neurons all across LIPd and LIPv. This pattern of intrinsic connectivity is in stark contrast to the patterns seen when the injection is only placed in LIPd.

**Fig. 7:**
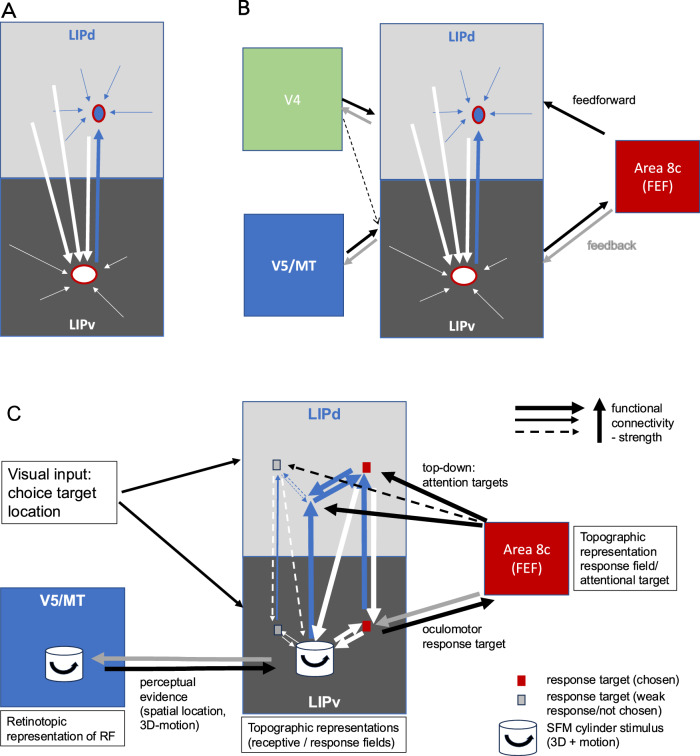
Summary of LIP intrinsic connectivity, the wider cortical network and the proposed circuit for processing perceptual decisions about 3D motion in macaques. **A** This diagram summarises the key findings of the intrinsic connectivity pattern between LIPd and LIPv from this study. LIPv, previously shown to be topographically organised^25^, sends a point-to-point projection to LIPd (blue arrows). In turn, each point in LIPv receives inputs from a number of different regions within LIPd (white arrows). Within both compartments, there is widespread connectivity. **B** Illustration of selected, previously established cortico-cortical in- and outputs to LIPv and LIPd. Extrastriate visual area V5/MT projects exclusively to LIPv, not LIPd, while ventral stream visual area V4 has a stronger projection to LIPd^9,13^. The frontal eye fields (FEF) send a feedforward-type projection to LIPd, and a feedback-type projection to LIPv^15^. These connections with FEF are reciprocal. **C** Illustration of the proposed processing scheme for perceptual decisions about 3D structure-from-motion stimuli, based on input of perceptual evidence represented in area V5/MT^49,50^ projected in a topographic fashion to LIPv. This input can convey perceptual signals about direction of motion and 3D depth localised to specific locations in the visual field. The intrinsic network between LIPv and LIPd can map one input (topographically mapped in LIPv) to other potential visual field locations through the connectivity loop with LIPd. The specific association between the sensory RF and saccade MF might be achieved gradually in a recurrent network. Interconnected with a feedforward connection into LIPd and receiving input from LIPv, FEF signals could support an attentional shift from stimulus to choice target representations and thus facilitate the association of the stimulus with a specific choice target. The map of planned saccade target locations in FEF is thus connected to the visual perceptual map in V5/MT through the circuitry in LIP. The specific associations of RF and MF in single neurons we found could be the product of the daily visual experience of the animals directing saccades to salient visual features and the specific training the animals underwent to learn to make perceptual decisions about 3D structure-from-motion objects. Arrows intrinsic connectivity: blue – projection to LIPd injection site, white – to LIPv injection site; arrows inter-area connectivity: black – feedforward, grey – feedback.

These anatomical wiring patterns clearly distinguish two operational compartments. The sparseness of the LIPv to LIPd projection is similar to that of early and mid-level visual areas and would be suitable to maintain a spatial map of visual field positions for visual stimuli and saccade targets. LIPv is the part of LIP that received direct, but sparse input from extrastriate visual area V5/MT^9^, so is in a position to receive organised visual information about moving and 3D stimuli for perceptual decision-making or guiding eye movements (Fig. 7). This is consistent with prior evidence of a topographically organised LIPv^9,25,26^ and an oculo-centric representation of the perceptual decision formation^37^. Putting these anatomical findings into context of the cortico-cortical connectivity pattern of LIP^9,11–15^, we propose an operational circuit in LIP that can link precise spatial and object visual information received from V5/MT and V4 to saccade and attentional targets conveyed through the circuitry with FEF (Fig. 7). We have here potentially two recurrent network circuits: one intrinsic to LIP between LIPv and LIPd and another one connecting LIP with FEF. FEF could convey attentional or oculomotor planning signals via a feedforward-type connection into LIPd and also receives decision-related information and signals for planning eye movements from topographically organised LIPv.

The anatomical circuitry is complemented at the cellular level by the neurophysiological properties of the LIP neurons we have mapped in this study. Visual, attentional or saccade planning selectivity of single neurons in area LIP has been described in a number of papers^9,18,23,38^, though individual studies put the emphasis on one type of property measured in a specific behavioural context (delayed saccade task for MF and fixation task or anesthetized recordings for RFs). The most common link between sensory RF and intentional MF is the reported response to a visual stimulus placed within the MF, describing RF and MF as overlapping (e.g. ref. ^7^). But some differences in MF and RF within the contralateral visual field have been indicated when the RF was probed with shapes^39^. On the basis of fMRI and neurophysiological evidence, it has been proposed that LIPv contains two overlaid or interdigitating topographic maps for visual attention and saccade planning^27^. Using stereotactically targeted, electrophysiological recordings, we show now that this pattern is evident in single neurons. Single LIP neurons can have a sensory RF and a motor MF that are a spatially distinct from each other. As our examples show, many LIP neurons have clearly defined RFs and MFs. While some of these can span large parts of the visual field and they can also overlap (see Supplementary Fig. 6), there are clear examples with not just distinct response peaks for RF and MF but completely separate fields (Fig. 5 and Supplementary Fig. 6). Our data suggest that the latter are localised to LIPv. But of course, the border between LIPd and LIPv is difficult to determine in neurophysiological recordings and its recording depth might vary on different vertical penetrations. A larger dataset in connection with histology and linear array recordings are required to confirm this.

The demonstration of spatially separated RFs and MFs for the same neuron offers a simple mechanistic explanation not just for seemingly contradictory studies on perceptual decision-making and their interpretation of the role of area LIP in sensorimotor processing^7,18,38,40,41^. They also provide a potential neural mechanism for how perceptual evidence could be processed and gradually associated with a specific decision response in the LIP circuitry. Some evidence for this stems from a recent inactivation study by Zhou and Freedman^7^ for overlapping RFs and MFs in LIP. We show now that there are other sets of neurons with different spatial configurations, particularly in more ventral parts of LIP, which will respond differently depending on where the stimulus to be judged and the response targets are placed. The RF and MFs we find suggest that responses of these neurons are generated by the specific combinations of sensory input and the saccade this instructs. These associations of disparate MF and RF tile the visual field.

How are these functional associations circuits formed and what is their role? We propose that this association between specific sensory information in the RF and the saccade planning to distinct MFs develops because of the visual diet of animals, for instance directing their gaze in response to moving stimuli. Of course, the behavioural training in perceptual decision-making undergone by animals in this study should also shape this functional circuitry. The particular configurations we recorded might be a direct result of this training. During perceptual decision-making, the anatomical processing loops within LIP and with FEF could provide the neural substrate for recurrent processes that generate specific associations between sensory RFs and saccade related activity (Fig. 7), which would be measured in rising activity during decision formation and the sustained activity in a delay before a response in the form of an eye movement. A critical test for this hypothesis will require the determination of the temporal order of neural events and their cross-correlations across LIPd and LIPv neurons during perceptual learning and decision-making. These need to be established with high-density recordings in behaving primates.

In summary, activity of LIP neurons has been linked to cognitive functions, like perceptual decision-making about random dot motion stimuli^41,42^ and categorisation of such visual stimuli^7,43^. Recent studies also suggest that the shifting perceptual evidence or attentional signals in such tasks are dynamically represented within LIP^6,37,44^. As neuronal mechanism, a common circuitry framework might serve that dynamically associates relevant sensory cues with disparate locations representing upcoming attentional or saccade targets. Our anatomical and neurophysiological results delineate such a neural circuit within LIP that can flexibly link visual object information to a cognitive or behavioural target.

## Methods

### Animals

Combined neurophysiology and behavioural experiments were performed in two adult male macaque monkeys (*Macaca mulatta*, M133, M134) weighing 13–17 kg. For each animal, structural MRI scans (MPRAGE 0.5 × 0.5 × 0.5 mm isotropic; blackbone; on a Siemens 3 T scanner) were obtained under general anaesthesia. Based on the MR scans, animals were implanted with a titanium fixation device (Graymatter, USA) and a stereotactically placed, vertical titanium recording chamber (Thomas RECORDING GmbH, Germany) was mounted over the craniotomy above posterior parietal cortex to allow recordings parallel to the parasagittal and coronal planes and orthogonal to the horizontal plane.

For the histological study, six adult macaque monkeys (*Macaca mulatta;* 4 females, 2 males, age range 4.8–12.4 years; weight 6.2–14.1 kg) were included (Table 1). All procedures were carried out under general anaesthesia. For each animal, a structural MRI scan (MPRAGE 0.5 × 0.5 × 0.5 mm isotropic; on a Siemens 3 T Scanner) was obtained. Five animals received a craniotomy and an MR-compatible chamber (Rogue Research Inc., Canada) centred on the lunate sulcus of the left hemisphere in a separate procedure prior to the injection. All procedures conformed to United Kingdom Home Office regulations on animal experimentation and to the EU Directives of the European Parliament and the Council in force at the time.

**Table 1:** Details of animals and injections in the histological study

NHP ID	Sex	weight (kg)	Age (years)	Transport (h)	Tracer	Amount	Approach	LIP	Injection site
**M127**	M	14.1	12.4	113	CTb (1%)	120 nL	posterior	LIPd/v border	Layers 1/2/3A
**M128**	F	6.2	4.8	186	CTb (1%)	80 nL	posterior	LIPd	Layers 3B/4/5
**M129**	F	6.2	4.8	164	FG (2%)	100 nL	posterior	LIPd	Layers 3A/3B/4
**M131**	F	7.4	6.1	234	CTb (1%)	80 nL	posterior	LIPd	Layers 3B/4
**M132**	F	6.3	6.3	278	CTb (1%)	80-100 nL	posterior	LIPv	Layers 5/6
**M140**	M	8.8	6.2	87	CTb (1%)	80 nL	dorsal	LIPv	Layers 1–5

Sex is given as M for male and F for female. Transport gives the survival time of the animal post-injection which was available for active transport of the tracers. If the amount of tracer is given by a range, the fluid meniscus in the glass pipette was between the two marker lines. Approach describes the trajectory of the electrode, either from posterior or entering dorsally. LIP gives the subdivision where the injection site was placed (d-dorsal; v-ventral). Injection site describes the layers where we found discernible tracer deposits from the injection. We would need more animals and even smaller injection sites to systematically assess the impact of layer on the pattern of connectivity.

### Awake neurophysiology experiments and analysis

Monkeys M133 and M134 were trained to maintain fixation on a binocularly presented visual stimulus and to perform a delayed saccade task while their eye movements were video tracked (SensoMotoric Instruments [SMI], Germany). They received fluid rewards for correct responses or maintaining fixation and their daily fluid intake was controlled.

Visual stimuli were displayed binocularly using a Wheatstone stereoscope comprising a set of mirrors and a pair of CRT monitors (Eizo FlexScan F78, UK) at a viewing distance of 84 cm. Monitors had a mean luminance of 42 cd/m^2^, a resolution of 1600 × 1200 pixels, a screen frame rate of 85 Hz, and covered 26.7° × 20.1° of the subject’s visual field. Quadro K2200 (NVIDIA) video cards in multi-core Intel processor computers running Ubuntu 14.04 (Linux kernel 4.4.0-124-lowlatency) were used to drive the monitors. Stimuli were programmed using PsychToolbox (3.0.14) running in Matlab R2015b (The Mathworks, USA). All stimuli were presented on a mid-grey background, using OpenGL alpha-blending to obtain sub-pixel resolution.

Animals performed a fixation task for mapping the sensory receptive field and a delayed saccade task for mapping the motor response fields^36,45^. For the fixation task, monkeys received a fluid reward for maintaining fixation on a central white fixation point (0.2° diameter, circular dot) for 1 s (RF mapping and direction tuning). If the gaze fell outside of the fixation window (radius 1°–2°), the trial was aborted. For all neurons, we mapped visual receptive fields with a patch of coherently moving black-and-white dots (0.2° diameter) while the monkey maintained fixation. The position, speed, direction, and circular size of the patch of moving dots on the computer screen was adjusted interactively to maximise neuronal responses of the isolated single unit. For a large fraction of LIP neurons (67 out of 111), we also mapped the receptive field quantitatively by probing the visual field with a circular patch of moving, black and white dots (dot diameter 0.2°; patch radius 3°; random motion or if little response also coherent motion). The patch was randomly presented centred at a combination of one of four y-axis positions (−6°, −3°, 3°, 6°) and six x-axis positions (−9°, −6°, −3°, 3°, 6°, 9°). For all neurons, we also measured direction tuning in the mapped receptive field using a patch of black and white, coherently moving dots matched in patch size and dot speed to the receptive field (randomly interleaved: directions of 0°, 60°, 120°, 180°, 240°, 300°, and blank screen), which we used to test for a significant response to the visual stimulus if no quantitative RF map was available (*n* = 44).

For the delayed saccade task: after fixating a white central fixation point (0.2° diameter) for 1 s, a white saccade target dot (0.2° diameter) appeared in the periphery. The saccade target was randomly presented at one of 5 y-axis positions (−6°, −3°, 0°, 3°, 6°) and in either 2 or 7 x-axis positions (−6°, 6° or −9°, −6°, −3°, 0°, 3°, 6°, 9°) from the centre of the screen. When the fixation point was extinguished after a further 1 s (“go” signal), the animal was required to make a saccade to the target (radius 1.5°, 2.0° or 2.5° [2 sessions]) within 300 ms to obtain a fluid reward. To calculate the delay activity, we used the 1 s period when both fixation point and saccade targets were on and before any saccade was initiated.

Neurophysiological recordings in area LIP were carried out through a vertical chamber using a smokestack with guide grid (1 mm spacing; Crist Instruments Co. Inc., USA), which was centred stereotactically over LIP using MRI guidance (Brainsight, Rogue Research Inc.). This allowed us to systematically map neural response properties along dorso-ventral penetrations in a circular window with a diameter of 15 mm). We recorded single units with tungsten microelectrodes coated with polyimide tubing (0.8 –1.2 MΩ impedance at 1 kHz; FHC, MicroProbe Inc., USA) inserted into the cortex through a short guide tube and advanced using a hydraulic microdrive (MO-97, Narishige Group, Japan). Electrical signals were filtered, amplified, and displayed through visual and audio monitors, and stored to computer disk (Blackrock recording system, Blackrock Microsystems LLC, USA). Binocular eye movements of the animal were recorded with an SMI eye tracker and the iView X system programme (SensoMotoric Instruments [SMI], Germany). Offline spike sorting was carried out with Blackrock Offline Spike Sorter (BOSS) (Blackrock Microsystems LLC, USA) and were subsequently analysed using software tools developed in MATLAB (MathWorks, USA).

**Fig. 8:**
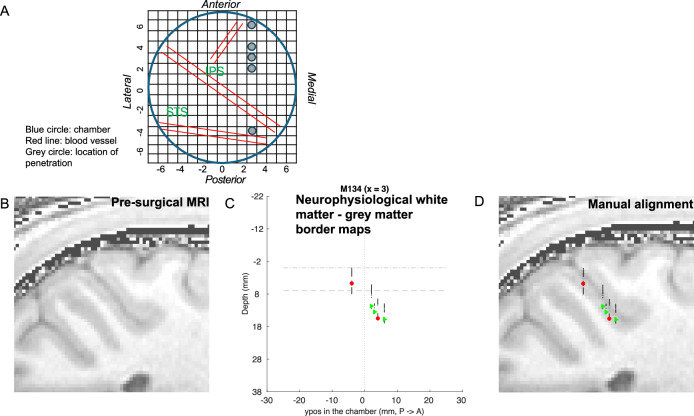
Positioning and alignment of neurophysiological penetrations. **A** shows a view into the vertical chamber (blue circle) implanted on the skull of one animal (M134). It is viewed with a superimposed, schematic grid as used for stereotactic penetrations in the neurophysiological experiments. The filled grey circles indicate the locations of the example penetrations/cells in the following panels. **B** shows a sagittal slice from the pre-surgical MRI. **C** Neurophysiological white matter and grey matter maps along a posterior-to-anterior row of penetrations in one parasagittal plane (x = 3 in the chamber grid) and depth from entry into the cortex. Red-filled circles and green-filled triangles indicate the recorded cells (convention as in Figs. 5 and 6: red – RF and MF both contralateral; green – RF and MF in different visual hemifields). On each penetration, we identified borders of grey matter and white matter by background neuronal sound and spiking activity. We plotted a black line indicating clear grey matter and a blank indicating the white matter (no response of cells and background sound). The dashed horizontal lines represent entry to cortex and putative LIPd/LIPv border respectively. **D** The black-blank lines from (**C**) were aligned with the borders between grey matter and white matter in the pre-surgical MRI shown in (**B**).

Area LIP was identified by carefully recording electrode depth and grey matter/white matter boundaries every 200 μm on each penetration. These were aligned by x- and y-position on the guide grid to the structural MRI scans taken from the animals before chamber implantation. We imported structural MRI data obtained previously from the individual animal into Matlab (command *read_avw*) and extracted contours with *contourslice* to indicate the tissue boundaries. First, we checked the fit of penetrations for each parasagittal plane, subsequently also in the horizontal plane across all penetrations. We found the best fit for the neurophysiological data to the MRI structural scan for each monkey by adjusting the overall positioning of the centre of the guide grid over cortex by 3–5 mm in the horizontal plane. This was necessary due to potential alignment changes during the surgery to fit the custom-made Titanium chamber (Thomas Recording, Germany) to the skull. Once aligned, the absolute depth of individual penetrations could be adjusted by 1–3 mm (necessary due to the use of different lengths guide tubes for penetrating the dura). Recorded grey matter/white matter boundaries for each penetration and horizontal relationship between penetrations were preserved as recorded (Fig. 8). Response properties on all included penetrations showed at least qualitatively a clear delayed saccade response in the target structure.

To establish the sensory receptive field (RF), we tested for significant variation in evoked response to the presented stimuli in the quantitative mapping task (after deducting firing rate during the preceding fixation without a stimulus; ANOVA, *p* < 0.05). For the sensory evoked response, we used the firing rate for the time (1 s) from stimulus onset to stimulus offset for correct fixation trials. If no quantitative RF mapping was collected, we used the responses from the direction tuning measurements, comparing RDK stimulus trials against blank screen (t-test *p* < 0.05). To examine firing rates for the saccade delay period (MF), we subtracted the firing rate of the preceding fixation period from the firing rate of the delay period (1 s) from target onset to fixation offset. We carefully excluded all saccade related activity. We tested whether there were significantly different responses for the different saccade target locations (ANOVA, *p* < 0.05). We identified the saccade target region that led to the strongest responses during the delay period as the saccade response field of the cell. The distance between RF and MF was calculated as the distance (in degrees of visual angle) between the centre locations for the two probe stimuli (visual stimulus and saccade target) which elicited the strongest response. To smoothly map the mean firing rate across the sampled visual space, we used Matlab commands (*meshgrid*, *interp2*, *surf*) to linearly interpolate between the centre positions of sample stimuli. The resultant 3D surface is viewed from above, with the colour scale indicating mean firing rate above baseline as described above. For unsmoothed images see Supplementary Fig. 5.

### Tracer injections

Tracer injections were placed either in a recovery procedure or in a five-day terminal procedure, all under general anaesthesia. For induction, animals received ketamine (7.5 mg/kg), midazolam ([Hypnovel], 0.1 mg/kg), xylazine (0.125 mg/kg) (i.m.) as well as atropine sulphate (0.05 mg/kg) (i.m.). Animals were intubated and artificially ventilated (2–3% Sevoflurane). The head was placed in a stereotaxic frame. Non-invasive blood pressure, heart rate, electrocardiogram, oximetry, and body temperature were monitored throughout. Hartman’s solution (Ringer’s lactate solution) was given through a cannulated saphenous vein. For the animal without a chamber (M140), a craniotomy was made. For the visual stimulation, contact lenses of +3D were placed on both corneas and spherical lenses placed to focus the eyes on a screen at 1.14 m.

Under general anaesthesia (~2.5% Sevoflurane by inhalation or 0.006 mg/kg/hr Sufentanil i.v. + 0.5% Isoflurane by inhalation), we made a small durotomy and advanced a glass capillary tube containing the retrograde tracer attached to a tungsten electrode (Microprobe Inc., impedance 0.65–0.75 MOhms) (Fig. 1A). Trajectory and target site for the injection were identified by a combination of MR-based guidance (Brainsight, Rogue Research Inc., Canada), stereotactic coordinates and neuronal recordings on approach. For LIPd injections, a posterior approach was chosen to insert the electrode into cortex with an angle of 5–20° over the horizontal plane 1–2 mm anterior to the lunate sulcus and 10–11 mm lateral to the midline. For LIPv, we chose either a posterior or a dorsal approach entering anteriorly to the intraparietal sulcus (IPS). We recorded grey matter/white matter boundaries as we advanced the electrode and mapped multi-unit visual receptive fields.

Five monkeys (M127, M128, M131, M132, M140) were injected with 80–120 nl retrograde tracer Cholera Toxin subunit b (CTb, 1% low salt solution, List Biological Labs, USA), one female monkey (M129) was injected with 100 nl retrograde tracer Fluorogold (FG, Fluorochrome LCC) (Table 1). After 87–278 h, animals were perfused and the tissue was processed.

### Histology

After transcardial perfusion with 0.9% heparinised PBS and 4% Paraformaldehyde, the left hemispheres were removed and cryoprotected in 10%, 20% and finally 30% sucrose in 0.1 M Phosphate Buffer. Tissue was sectioned parasagittally at 50 µm on a freezing stage microtome. A one-in-five series each were stained for Gallyas^46^ and Nissl^47^ to determine the boundary between LIPd and LIPv and to adjacent areas. Nissl-stained sections were also used for discrimination between different cortical layers. Another one-in-five series was reacted for CTb staining or FG staining. All reactions were carried out on free-floating sections to maximise penetration. If reactions were not carried out immediately, sections were stored in cryoprotectant at −20 °C. Sections were treated with a peroxidase blocker to reduce background signal. Next, sections were incubated for 16–20 h at 4 °C in peroxidase blocker containing either primary antibody against Cholera Toxin subunit b (List Biological laboratories) diluted 1:9000 for sections stained for CTb or rabbit anti-fluorogold (Fluorochrome LCC) diluted 1:1000 for sections stained for FG. This was followed by incubation for 90 min at room temperature with secondary antibody anti-goat or anti-rabbit IgG Biotin (Sigma) diluted 1:400 or 1:300, respectively. All sections were treated with ABS (Vector Elite) for 60 min at RT before they were reacted with SigmaFast^TM^ DAB for 5 min at room temperature to visualise stained cells. Finally, sections were mounted on gelatinised slides, air-dried at 37 °C, dehydrated and cover-slipped with DPX (Merck).

### Histological analysis

We quantified the distribution of retrogradely labelled cell bodies in LIP using a computerised microscope (Neurolucida, Microbrightfield Ltd; Matlab, MathWorks) with the histological sections. The outline of the intraparietal sulcus and the grey matter/white matter boundary were drawn at 2x magnification. Locations of CTb or FG labelled cells were registered by marking cell bodies at high magnification of x10. FG- and CTb-labelled cells showed distinct patterns of label in the cell body allowing good identification. We analysed a complete one-in-five series of parasagittal sections through LIP for each monkey. The most lateral and medial sections which only showed a low number of labelled cells or in which border discrimination of LIP was not reliably possible were excluded from the quantitative analysis. For one hemisphere stained for CTb (M127; injection at the LIPv/d boundary), the injection site included layer I leading to a high number of labelled cells in layer I. These cells were excluded from the analysis in order to compare the labelling patterns across hemispheres.

All injection sites were neurophysiologically identified at the time of injection. The effective tracer up-take zone is thought to be the darkest area around the injection site in which labelled cells are not discernable under bright illumination and high magnification (x10)^48^. Such a core injection sites could be confirmed histologically in all animals. In one animal (M132L), the visible tracer deposit was very small and only apparent in layers 5/6 of one section of the 1-in-5 series (see Table 1). Injection sites were usually small, around 500–800 μm in diameter and well circumscribed with some lighter diffusion of tracer around it. We have previously confirmed that CTb is not taken up by fibres of passage^28^, so the presented results show specific grey matter connectivity.

Interleaved sections stained for Gallyas or Nissl were examined for changes in myelination and cell patterns in LIP and adjacent areas at a magnification of 10x to discern borders between areas and subdivisions of LIP. In most cases, the investigation of nearby Gallyas sections was sufficient to discern the border between LIPd and dorsal area 7a. LIPd showed two prominent bands of Baillarger and lighter myelination than 7a^9–11^. In the few sections, in which this criterion was not clear, we also examined Nissl-stained sections for differences in cell densities and cell arrangements between 7a and LIPd^9,11^. The border between ventral and dorsal LIP was readily distinguishable by differences in myelination patterns^9–11^ (Fig. 1B). The discrimination of the border between LIPv and the adjacent ventral intraparietal area (VIP) was based on the decrease in myelination in VIP. In some sections, this was supplemented by examining Nissl-stained sections for differences in cell densities and organisation^10^.

The border between layer 4 and layer 5 was outlined based on differences in cell density and appearance between layers in Nissl sections interleaved with the CTb/FG labelled sections using Neurolucida at 10x magnification. These outlines were overlaid on the CTb and FG labelled sections to determine the border of layers 4/5. In sections stained for CTb layer 4 hardly contained any labelled cells which made it easier to define the boundary to layer 5.

To examine the pattern of labelled cells across sections, we created cell density maps (Fig. 1F). We then adjusted the cross-section scale bar to the highest density of labelled cells found within LIP in this hemisphere (see Supplementary Fig. 1 for the potential effects of different normalisation methods). We analysed supragranular layers (2–4) and infragranular layers (5-6) separately and aligned the 2D plots across LIP sections.

### Reporting summary

Further information on research design is available in the Nature Portfolio Reporting Summary linked to this article.

## Supplementary information

Supplementary Information

Description of Additional Supplementary Materials

Supplementary data

Supplementary Movie 1

Reporting Summary

## Data Availability

The histological cell positions and boundaries are stored as text files. We make these available in data sheets that allow the visualisation for each section of the outline of LIP and the labelled cell positions. The neurophysiological, spike sorted data are available as Matlab arrays with spike counts for the different trials, sorted by animal, neuron, experiment and stimulus. We will make the data available on request with guidance on the formats and contextual information (e.g. stimuli; eye traces) to allow re-use.
