# Supplementary material for: Anatomical circuits for flexible spatial mapping by single neurons in posterior parietal cortex
Source: Commun Biol. 2025 Sep 9;8:1337. doi: 10.1038/s42003-025-08596-6 (PMC12420791; doi:10.1038/s42003-025-08596-6)
Supplement: Supplementary file 1 — Supplementary Information [file 42003_2025_8596_MOESM1_ESM.pdf]

Supplementary information

Supplementary Movie 1.

Animated 3D reconstruction of the pattern of labelled neurons in one brain. The movie shows an aligned 1-in-5 series of sections with Fluorogold(FG)-labelled cells after an injection into dorsal LIP (LIPd) of the left hemisphere of M129. FG-labelled cells are drawn in green with the bulk of labelled cells around the injections site in LIPd. A smaller cluster of labelled cells is visible just ventral and slightly medial to the larger cluster in ventral LIP (LIPv). A second injection site of a different tracer into extrastriate visual area V5/MT is depicted in red for orientation. The x-axis runs posterior-to-anterior, the y-axis ventral-to-dorsal and the z-axis lateral-to-medial.

Supplementary Data

This .xcel file contains the data for the graphs in Figure 5A, 5D, 6A, 6B and 6D.

Supplementary Figure 1.

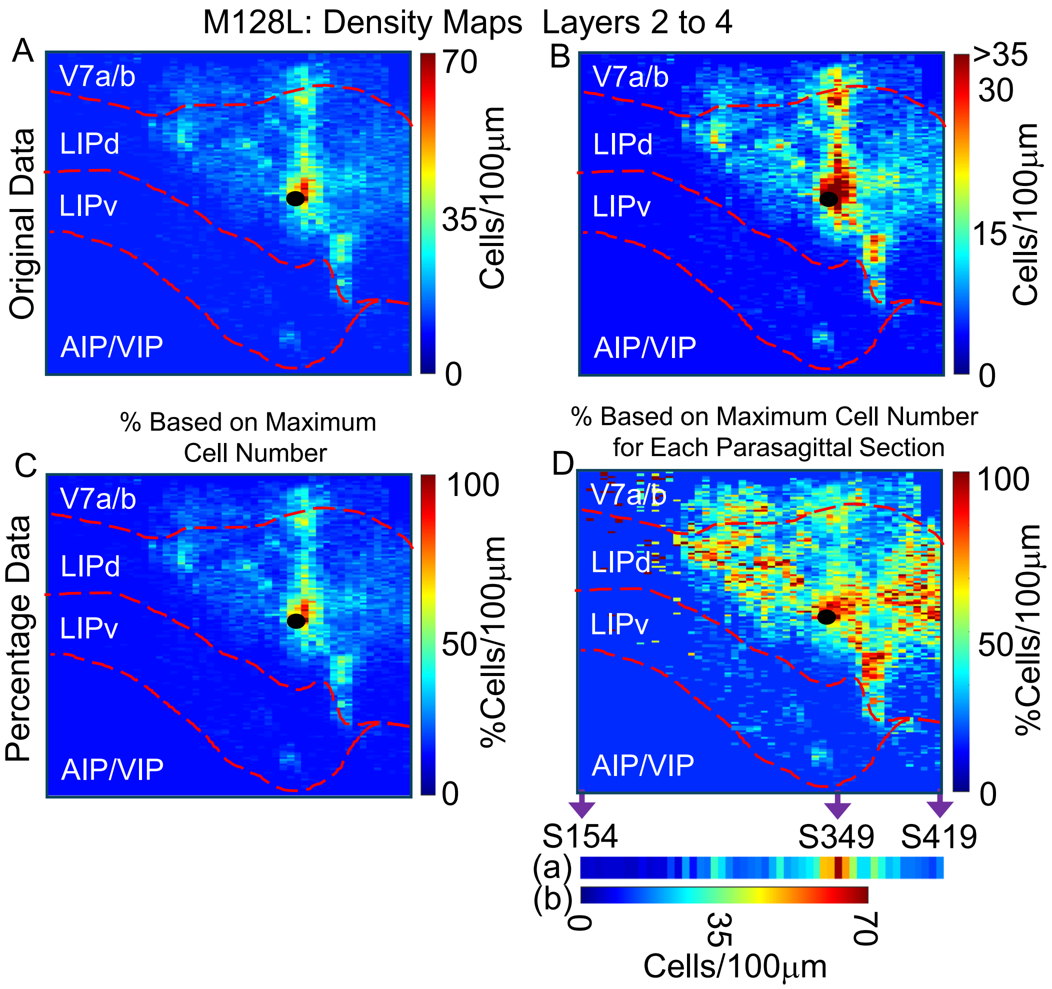

When normalising the presentation of cell densities, accurate number representations as well as visibility of patterns need to be balanced. This figure illustrates the effect of different choices for the presentation for our summary histology figures. **A.** This plot depicts the actual cell counts per bin. The numbers represent the cell count for a length of 100 μm measured dorso-laterally along the layers 2-to-4 or 5+6 in each section (see also Fig. 1 main paper). **B.** In this plot, like in the paper, we condensed the range of the colour scale indicating cell density, so that the highest values for this LIP are all depicted by red (e.g. > 30 cell/100 μm). This allows us to visualise some of the lower densities of labelled cells. **C.** If we normalised cell densities per 100 μm by the peak density of label for the entire LIP, we would get a pattern equivalent to the actual cell count in A. We cannot anymore visualise the presence of labelled cells in sections with lower densities. **D.** If we were to normalise by peak count for each section, we would see a distribution that is no longer representative of the true distribution of cell densities across LIP as a whole, and even very low cell counts would show up if in a section with few labelled cells. The colour bar (a) depicts the peak density in each parasagital section; meaning of the colourscale used in (a) is given in (b).

Supplementary Figure 2.

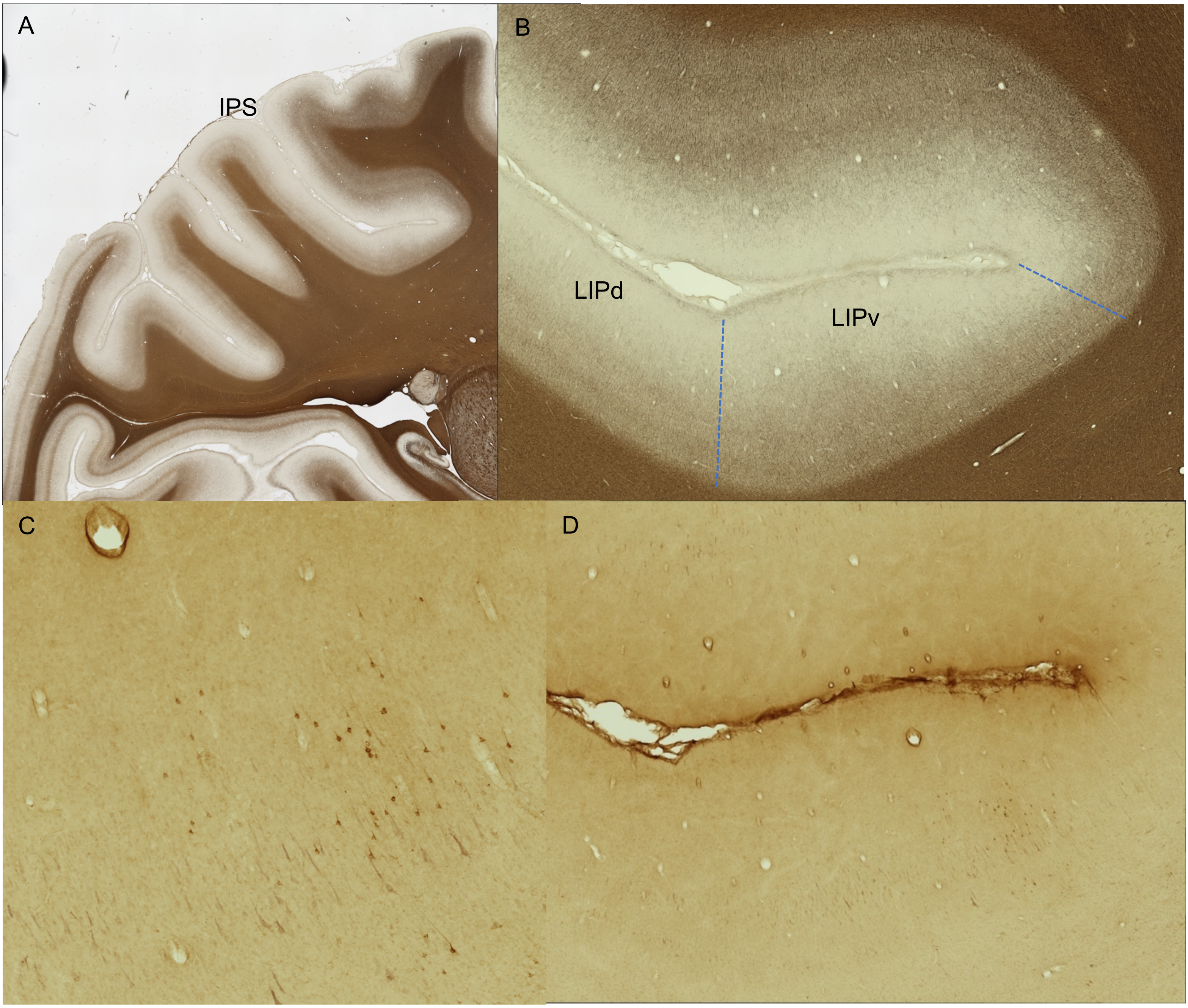

Parasagittal sections from one hemisphere after injection of retrograde tracer CTb into LIPd (M128L). **A.** This section (341) was Gallyas-stained for myelin. It shows the posterior part of the brain with primary visual cortex (identifiable by the Stripe of Gennari) and on the right of the section the intraparietal Sulcus (IPS) with LIPd and LIPv. **B.** Larger image of the section from (A) showing the border between LIPd as identified by two light bands of myelin in the lower cortical layers, and LIPv, with the denser, more diffuse myelin pattern. **C.** High resolution image of the cluster of labelled neurons in LIPv in a nearby section (339, 100 µm away). Labelled cells on the right half of the image show dark granulated label inside the clearly discernable cell bodies. The ghostlike, pale shapes towards the left, do not contain CTb label. **D.** Lower magnification image of the same section, showing the same portion as the nearest myelin-stained section depicted in (B).

Supplementary Figure 3.

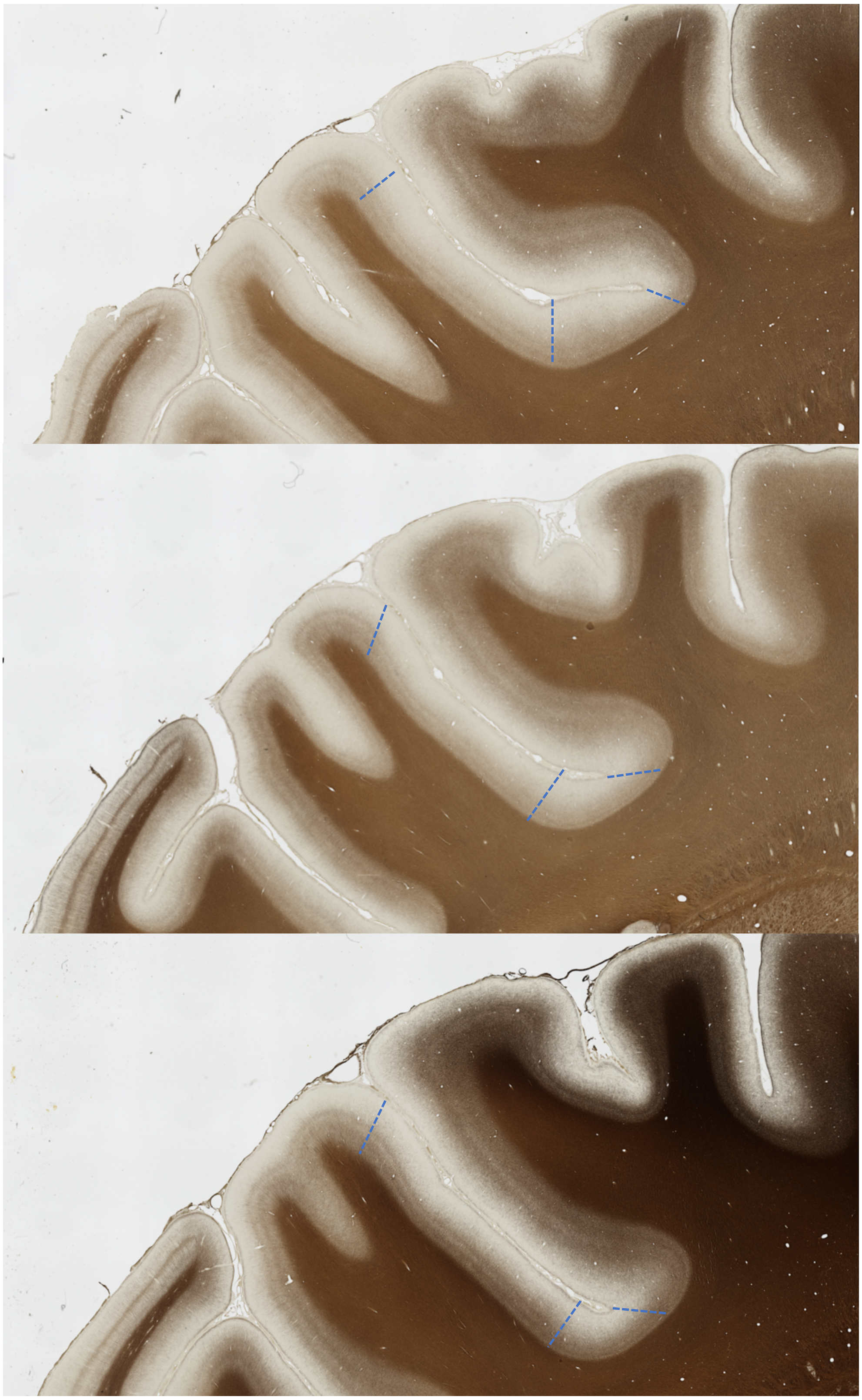

M128L
section 341

LIPd – distinct pale myelin double band

LIPv – diffuse, thick, myelin across layers 3-6

M128L
Section 361

LIPd – distinct myelin double band

LIPv – more diffuse myelin label across lower layers

M128L
Section 371

LIPd – distinct myelin double band

LIPv – more diffuse myelin label across lower layers

The three panels show parasagittal histological sections stained for myelin to determine the broders of LIPd and LIPv for one animal (M128): **Top:** Near the injection site. **Middle and bottom:** Ca. 2 mm medial, near the edge of identifiable LIPv.

Supplementary Figure 4.

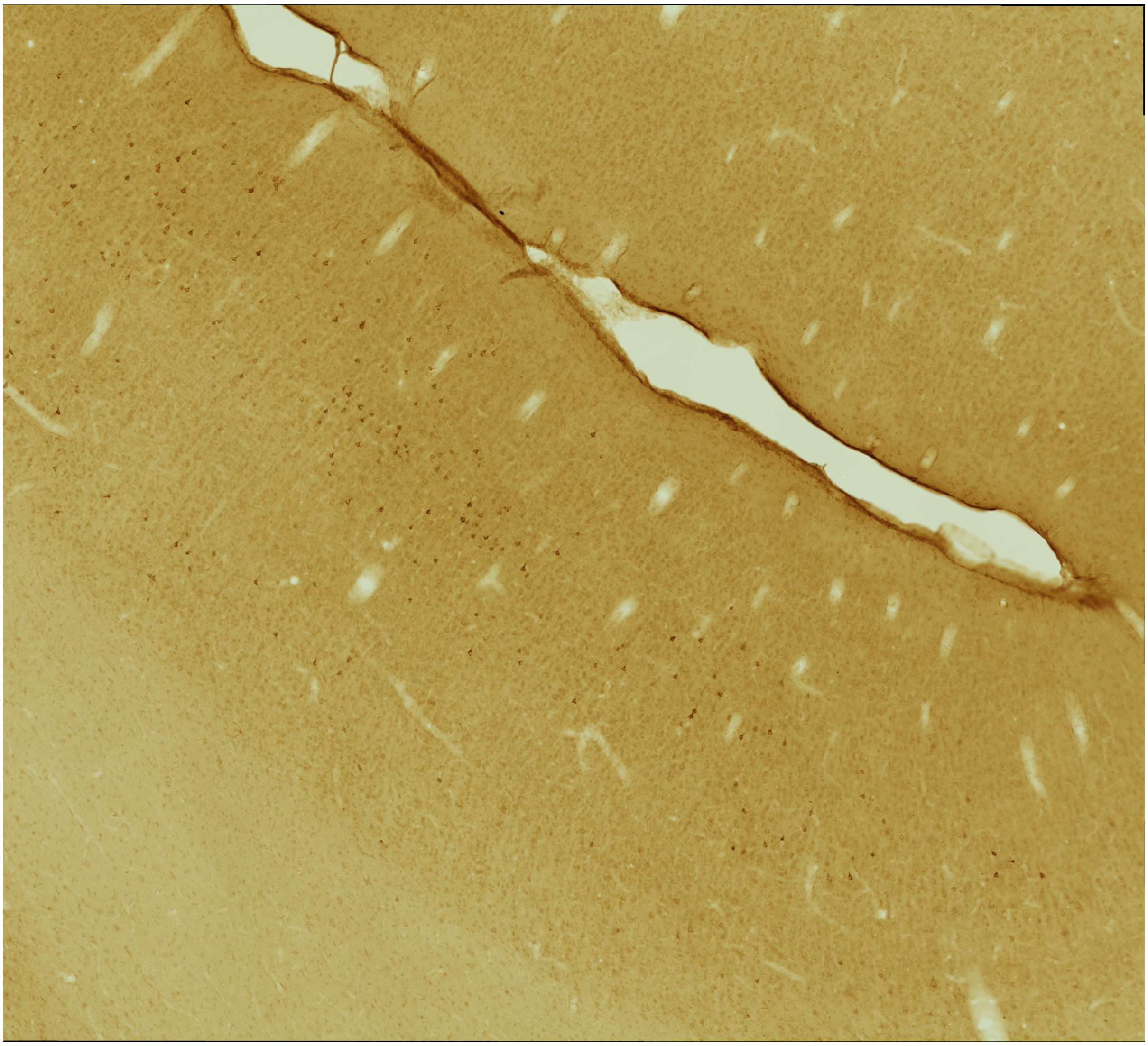

Example parasagittal section (366) for hemisphere M132L after a very small injection of retrograde tracer CTb into LIPv. The labelled cells in LIPv near the bottom of the sulcus are clearly discernible and wide-spread.

Supplementary Figure 5.

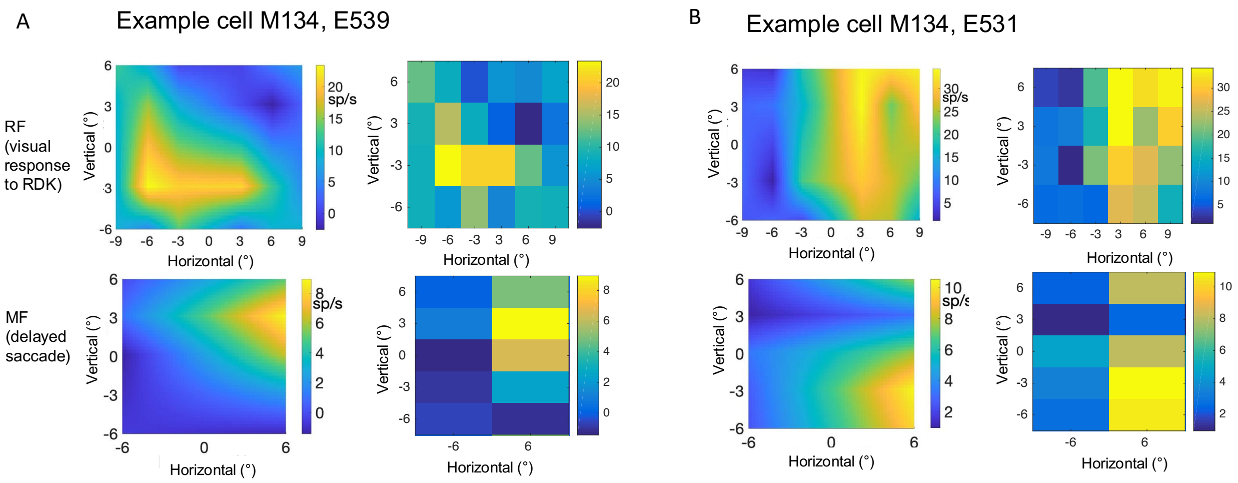

This figure shows the RF and MF plots for the two example cells (A. M134 E539 and B. M134 E531) in Fig. 5 of the paper. For each cell, the smoothed plots (see Methods) are shown on the left and the corresponding non-smoothed firing rate plots directly next to them on the right.

Supplementary Figure 6.

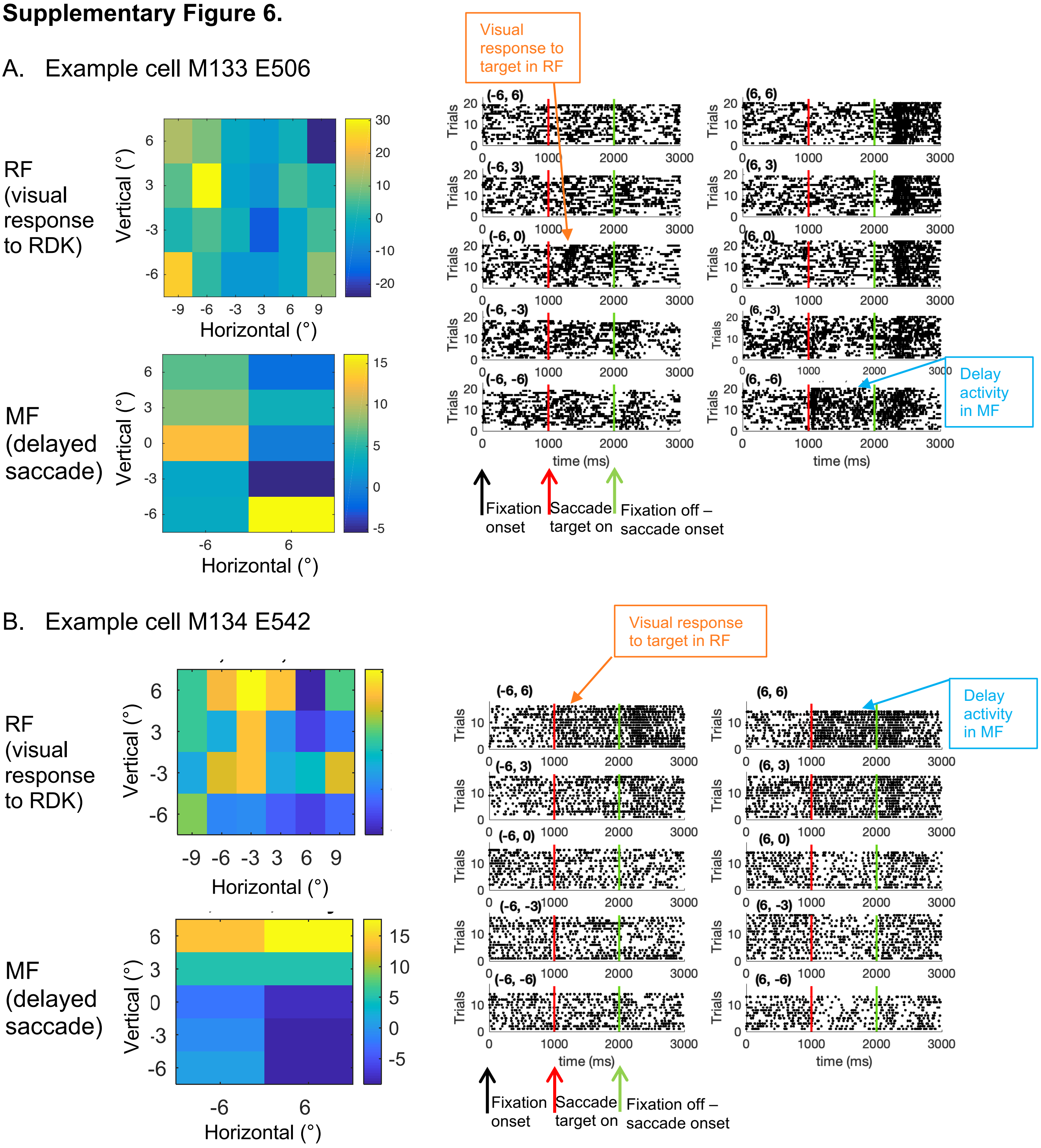

In the style of Fig. 5 of the main paper, we show two more example cells (A + B), each from a different monkey. **A.** This cell shows a distinct sensory RF and MF delay activity. Please note that the visual response will also light up in the MF mapping plot on the left, as is shown here. But the peak response for the delay period is clearly separate. **B.** One example cell with closer, overlapping MF and RF.
