# Supplementary material for: Anatomical circuits for flexible spatial mapping by single neurons in posterior parietal cortex
Source: Commun Biol. 2025 Sep 9;8:1337. doi: 10.1038/s42003-025-08596-6 (PMC12420791; doi:10.1038/s42003-025-08596-6)
Supplement: Supplementary file 2 — Description of Additional Supplementary Materials [file 42003_2025_8596_MOESM2_ESM.pdf]

Description of Additional Supplementary Files

File name: Supplementary Data

Description: the numerical source data for the graphs.

File name: Supplementary Movie

Description: Animated 3D reconstruction of the pattern of labelled neurons in one brain. The movie shows an aligned 1-in-5 series of sections with Fluorogold(FG)-labelled cells after an injection into dorsal LIP (LIPd) of the left hemisphere of M129. FG-labelled cells are drawn in green with the bulk of labelled cells around the injections site in LIPd. A

smaller cluster of labelled cells is visible just ventral and slightly medial to the larger

cluster in ventral LIP (LIPv). A second injection site of a different tracer into extrastriate visual area V5/MT is depicted in red for orientation. The x-axis runs posterior-toanterior, the y-axis ventral-to-dorsal and the z-axis lateral-to-medial..
