# Supplementary material for: Anatomical circuits for flexible spatial mapping by single neurons in posterior parietal cortex
Source: Commun Biol. 2025 Sep 9;8:1337. doi: 10.1038/s42003-025-08596-6 (PMC12420791; doi:10.1038/s42003-025-08596-6)
Supplement: Supplementary file 5 — Reporting Summary [file 42003_2025_8596_MOESM5_ESM.pdf]

nature portfolio

Corresponding author(s): Kristine Krug

Last updated by author(s): Jun 29, 2025

Reporting Summary

Nature Portfolio wishes to improve the reproducibility of the work that we publish. This form provides structure for consistency and transparency in reporting. For further information on Nature Portfolio policies, see our Editorial Policies and the Editorial Policy Checklist.

Statistics

For all statistical analyses, confirm that the following items are present in the figure legend, table legend, main text, or Methods section.

n/a	Confirmed	
☐	☒	The exact sample size (*n*) for each experimental group/condition, given as a discrete number and unit of measurement
☐	☒	A statement on whether measurements were taken from distinct samples or whether the same sample was measured repeatedly
☐	☒	The statistical test(s) used AND whether they are one- or two-sided<br>*Only common tests should be described solely by name; describe more complex techniques in the Methods section.*
☒	☐	A description of all covariates tested
☐	☒	A description of any assumptions or corrections, such as tests of normality and adjustment for multiple comparisons
☐	☒	A full description of the statistical parameters including central tendency (e.g. means) or other basic estimates (e.g. regression coefficient) AND variation (e.g. standard deviation) or associated estimates of uncertainty (e.g. confidence intervals)
☒	☐	For null hypothesis testing, the test statistic (e.g. *F*, *t*, *r*) with confidence intervals, effect sizes, degrees of freedom and *P* value noted<br>*Give P values as exact values whenever suitable.*
☒	☐	For Bayesian analysis, information on the choice of priors and Markov chain Monte Carlo settings
☒	☐	For hierarchical and complex designs, identification of the appropriate level for tests and full reporting of outcomes
☒	☐	Estimates of effect sizes (e.g. Cohen's *d*, Pearson's *r*), indicating how they were calculated

Our web collection on statistics for biologists contains articles on many of the points above.

Software and code

Policy information about availability of computer code

Data collection	Histological data were drawn on a computerised microscope with the software Neurolucida (Microbrightfield Ltd).<br>Neurophysiological data were acquired using a combination of a Blackrock recording system and a custom suite of Matlab (Mathworks) based programmes (MET) which run the behavioural task, displayed the stimuli (based on Psychtoolbox 3.0.14) and integrated the eye traces (collected with an SMI Primate Eyetracker.
Data analysis	Histological analysis of cell density was carried out in Matlab (Mathworks) as described in Figure 1F.<br>Spike sorting was carried out with Blackrock Offline Spike Sorted (BOSS) (Blackrock Microsystems LLC, USA).<br>All further neurophysiological data analysis in Matlab (Mathworks).

For manuscripts utilizing custom algorithms or software that are central to the research but not yet described in published literature, software must be made available to editors and reviewers. We strongly encourage code deposition in a community repository (e.g. GitHub). See the Nature Portfolio guidelines for submitting code & software for further information.

Data

Policy information about availability of data

All manuscripts must include a data availability statement. This statement should provide the following information, where applicable:

- Accession codes, unique identifiers, or web links for publicly available datasets
- A description of any restrictions on data availability
- For clinical datasets or third party data, please ensure that the statement adheres to our policy

For our type of data, there are no standardised formats and repositories. We will make the data available on request with guidance on the formats and additional information (e.g. stimuli; eye traces) to allow proper usage. The histological cell positions and boundaries are stored in a large number of individual text files - we will make these available on request in .xcel files. The neurophysiological, spike sorted data are available as Matlab arrays with spike counts for the different trials, sorted by animal, neuron, experiment and stimulus.
In the Supplementary Data, we provide the numerical source data for the graphs.

Research involving human participants, their data, or biological material

Policy information about studies with human participants or human data. See also policy information about sex, gender (identity/presentation), and sexual orientation and race, ethnicity and racism.

Reporting on sex and gender	We do not include human data. We fully report the sex of the animals used. See specific section below.
Reporting on race, ethnicity, or other socially relevant groupings	We do not include human data.
Population characteristics	See above.
Recruitment	See above.
Ethics oversight	See above.

Note that full information on the approval of the study protocol must also be provided in the manuscript.

Field-specific reporting

Please select the one below that is the best fit for your research. If you are not sure, read the appropriate sections before making your selection.

☒ Life sciences ☐ Behavioural & social sciences ☐ Ecological, evolutionary & environmental sciences

For a reference copy of the document with all sections, see nature.com/documents/nr-reporting-summary-flat.pdf

Life sciences study design

All studies must disclose on these points even when the disclosure is negative.

Sample size	The sample size was determined based on previous experience with non-humanprimate studies and the expected effect size. For the histological studies, we used 6 animals, 3 per injection site. Since the results were clear and broadly comparable for each injection site, we did not carry out any more.<br>We used two animals for the neurophysiological studies carrying out multiple recordings with acute penetration in each of these.
Data exclusions	For the histological data, all brains with a valid injection into LIP were included.<br>For the neurophysiological data, we included all neurons with a completed experiment. Statistical exclusions for tuning criteria etc. are fully described in the paper
Replication	The neuroanatomical results for each injection site were replicated in 1-2 additional individuals. All replications are shown.<br>We completed neurophysiological recordings from 111 LIP neurons across two monkeys, which passed spike-sorting offline.
Randomization	We randomly interleaved the probed visual field positions, saccade target location or visual stimulus condition in each block for the neurophysiological recordings.
Blinding	As the injection site is visible in the histological analysis, blinding is not possible. We drew only clearly filled cell bodies and criteria were double checked by a 2nd experimenter.<br>The neurophysiological analysis of response properties is automatised in Matlab.

Reporting for specific materials, systems and methods

We require information from authors about some types of materials, experimental systems and methods used in many studies. Here, indicate whether each material, system or method listed is relevant to your study. If you are not sure if a list item applies to your research, read the appropriate section before selecting a response.

Materials & experimental systems

n/a	Involved in the study
☐	☒ Antibodies
☒	☐ Eukaryotic cell lines
☒	☐ Palaeontology and archaeology
☐	☒ Animals and other organisms
☒	☐ Clinical data
☒	☐ Dual use research of concern
☒	☐ Plants

Methods

n/a	Involved in the study
☒	☐ ChIP-seq
☒	☐ Flow cytometry
☒	☐ MRI-based neuroimaging

Antibodies

Antibodies used	For the histological analysis, we used standard, commercially available primary antibody against cholera toxin b subunit (List Biological laboratories) diluted 1:9000 for sections stained for CTb or rabbit anti-fluorogold (Fluorochrome LCC) diluted 1:1000 for sections stained for FG. This was followed with the standard, commercially available secondary antibody anti-goat or anti-rabbit IgG Biotin (Sigma) diluted 1:400 or 1:300.
Validation	https://listlabs.com/product/buy-anti-cholera-toxin-b-subunit-goat/<br>https://fluorochrome.com/antibodies/

Animals and other research organisms

Policy information about studies involving animals; ARRIVE guidelines recommended for reporting animal research, and Sex and Gender in Research

Laboratory animals	Rhesus macaques (macaca mulatta)
Wild animals	This study did not involve wild animals.
Reporting on sex	We report the sex of the macaques monkeys in this study. The histology involved two male and four female monkeys, the neurophysiological study was carried out in two males.
Field-collected samples	This study did not involved field-collected samples.
Ethics oversight	All animals procedures were approved by the United Kingdom Home Office Licences and conformed to the European Communities Council Directives in force at the time.

Note that full information on the approval of the study protocol must also be provided in the manuscript.

Plants

Seed stocks	This study does not involve plants.
Novel plant genotypes	See above.
Authentication	See above.
